# Selective versus multi-segmental decompression and fusion for multi-segment lumbar spinal stenosis with single-segment degenerative spondylolisthesis

**DOI:** 10.1186/s13018-019-1092-2

**Published:** 2019-02-12

**Authors:** Wei Sun, Cheng Xue, Xian-ye Tang, Hu Feng, Feng Yuan, Kai-jin Guo, Jie Zhao

**Affiliations:** 1 Department of Orthopaedic Surgery, The Affiliated Hospital of Xuzhou Medical University, 99 Huaihai Xi Road, Xuzhou, Jiangsu Province People’s Republic of China; 2 Department of Orthopaedic Surgery, Shanghai Ninth People’s Hospital, Shanghai Jiao Tong University School of Medicine, 639 Zhizaoju Road, Shanghai, People’s Republic of China

**Keywords:** Lumbar spinal stenosis, Multi-segment, Degenerative spondylolisthesis, Selective decompression with fusion

## Abstract

**Background:**

Lumbar spinal stenosis, often accompanied by degenerative spondylolisthesis, is one of the most common conditions in the elderly. Decompression and fusion is a well-accepted treatment for single-segment lumbar spinal stenosis with degenerative spondylolisthesis; however, the treatment for multi-segment lumbar spinal stenosis with single-segment degenerative spondylolisthesis (MLSS) remains controversial. The objective of this study is to compare the effectiveness of selective decompression and fusion to multi-segmental decompression and fusion for MLSS.

**Methods:**

A total of 42 patients suffering from MLSS who underwent surgery between June 2012 and January 2015 were included in this analysis. Of the 42 patients with minimum 3-year follow-up, 22 underwent selective decompression and fusion, and 20 patients underwent multi-segmental decompression and fusion. Age, gender, symptom duration, operative time, blood loss, the number of decompressed segment and fused segment, and complication were compared between the two groups. The visual analog scale (VAS), Oswestry Disability Index (ODI) and Short Form 36 (SF-36) were used to assess efficacy.

**Results:**

Operative time, blood loss, and the number of fused segment in multi-segmental decompression and fusion group were greater than those in selective decompression and fusion group (*P* < 0.01). The VAS, ODI, and SF-36 scores at 1-year follow-up and 3-year follow-up were significantly improved compared with those preoperatively in both groups (*P* < 0.01) but were not significantly different between the two groups at each time point (*P* > 0.05). There was no iatrogenic spinal instability in the decompressed segments in selective decompression and fusion group, while three patients developed postoperative instability at the adjacent segments above the fused segments in multi-segmental decompression and fusion group at 3-year follow-up.

**Conclusions:**

Selective decompression and fusion is a safe and effective method for the treatment of MLSS, with the advantages of shorter operative time, less blood loss, and more preservation of spinal motion segments when compared with multi-segmental decompression and fusion.

## Background

Lumbar spinal stenosis is one of the most common causes of neurogenic claudication and radicular leg pain. Lumbar spinal stenosis is often accompanied by degenerative spondylolisthesis, which commonly occurs in patients more than 50 years [1]. Decompression plus fusion is the well-accepted treatment for patients with single-segment lumbar spinal stenosis with degenerative spondylolisthesis compared with conservative treatment and decompression alone [2, 3]. However, when it comes to multi-segment lumbar spinal stenosis with single-segment degenerative spondylolisthesis (MLSS), the management remains controversial.

Some surgeons selected to perform extensive laminectomy to provide the clinical improvement, though extensive decompression may cause segmental instability. In order to reduce the incidence of iatrogenic spinal instability, address the symptoms of lower back pain, and ensure the long-term efficacy, fusion was recommended for the decompressed segments [4, 5]. In view of the problems related to multi-segment fusion, such as increased trauma, medical care costs, and adjacent segment degeneration (ASD) [6, 7], recently, other clinicians suggested that patients with MLSS should be treated with decompressive laminectomy at the stenotic segments and a single-level fusion only at the slipped segments [8]. However, besides laminectomy itself, performing a laminectomy above a fused segment may increase additional stress on the less stable segment, which may lead to iatrogenic segmental instability if fusion is not performed [9]. Furthermore, in clinical practice, it is sometimes challenging to ensure sufficient spinal stability on the premise of adequate decompression in the non-slipped, stenotic segments, especially in patients with developmental lumbar spinal stenosis combined with foramen stenosis.

With the emergence of the concept ‘precision medicine’ [10], many medical domains are changing from traditional extensive pattern to modern precise pattern. An increasing number of spine surgeons are pursuing precise diagnosis and treatment for patients. Precise decompression is advocated for the purpose of maximizing the clinical improvement and minimizing the risk of iatrogenic instability and the need for concomitant fusion. Selective decompression and fusion, referring to precise decompression and selective fusion, seems to be a viable alternative in the treatment of MLSS. However, to our knowledge, there are currently no studies that have evaluated the efficacy of selective decompression and fusion for MLSS. In this study, we therefore compared the clinical outcomes of selective decompression and fusion with those of multi-segmental decompression and fusion in the treatment of MLSS.

## Methods

The diagnostic criteria of lumbar spinal stenosis include clinical symptoms of neurogenic intermittent claudication, radiculopathy, and/or lower back pain, and confirmed lumbar spinal stenosis based on CT and MRI. Anatomic lumbar spinal stenosis without any symptom was not included in the study. We retrospectively reviewed all 191 patients with symptomatic multi-segment lumbar spinal stenosis (≥ 2 levels) who failed 3 months of conservative treatment and underwent decompression and fusion between June 2012 and January 2015. Of the 191 patients with multi-segment lumbar spinal stenosis, there were some patients with associated degenerative spondylolisthesis, and the others without degenerative spondylolisthesis. Only patients with associated single-segment degenerative spondylolisthesis who have completed a minimum 3-year follow-up were included in this analysis. Patients with metabolic diseases, severe osteoporosis, severe chronic medical comorbidities, or a history of previous lumbar spine surgery were excluded.

### Surgical procedure

The decisions of surgical procedure were made by the senior surgeons, either selective decompression and fusion (group S) or multi-segmental decompression and fusion (group M). Generally, the patients in group S were treated by means of precise and limited decompression according to the site and degree of spinal stenosis, such as hemilaminectomy, undercutting decompression of the lateral recess, or total laminectomy with preservation of the spinous process, interspinous, and supraspinous ligaments. At the slipped segments, first, laminectomy and facetectomy were performed to acquire adequate decompression, pedicle screws and rod were used bilaterally, and then the discectomy and endplate preparation were performed. The height of disc space was restored by sequentially distracting the disc space and then the disc space was filled with morselized bone from the laminectomy and facetectomy. Finally, the suitable cage was inserted obliquely. The decompressed stenotic segments that might develop segmental instability due to resection of more than 50% of the facet joint were performed interbody fusion in the same way. In group M, extensive decompression (a standard laminectomy and foraminal decompression), pedicle screw fixation, and interbody fusion (using the method mentioned above) were performed at the slipped segments and all the decompressed segments.

### Assessment of clinical and radiological outcomes

Gender, age, symptom duration, stenosis segment, listhesis segment, the mean number of decompressed segment and fused segment, operative time, blood loss, and complications were compared between the two groups. All patients were scheduled for follow up at 3, 6, and 12 months postoperatively and annually thereafter. The primary outcomes for clinical assessment were the visual analog scale (VAS, which ranges from 0 to 10, with higher scores indicating more severe pain), Oswestry Disability Index (ODI, which ranges from 0 to 100, with higher scores indicating more severe disability related to back pain and leg pain), and physical function, general health, and mental health domains of the Short Form 36 (SF-36, which ranges from 0 to 100, with higher scores indicating less severe symptoms).

X-ray imaging was used to assess fusion status (the signs of solid fusion include continuous intervertebral bone bridge formation and angle change of the infused segments <4° in flexion-extension radiographs of the lumbar spine [11]), stability of the operative segments and the adjacent segments (the signs of instability include horizontal displacement of the adjacent vertebrae > 4 mm or angle change > 10° in flexion-extension radiographs), and other complications, such as hardware loosening or breakage, or fusion cage migration.

### Statistical analyses

Statistical analysis was performed with SPSS 17.0 (SPSS Inc., Chicago, IL, USA). The chi-square and Fisher exact test was used to compare the gender, stenosis segment, listhesis segment, and the incidence of complications between the two groups. Student’s *t* test was used to compare the age, symptom duration, the mean number of decompressed segment and fused segment, operative time, and blood loss between the two groups. A paired *t* test was used to compare VAS, ODI, and SF-36 scores preoperatively and postoperatively. *P* < 0.05 was considered statistically significant.

## Results

Of the 191 patients with multi-segment lumbar spinal stenosis, there were 56 patients with associated single-segment degenerative spondylolisthesis. Of the 56 patients, 2 patients with diabetes and 1 with severe cerebrovascular diseases were excluded. Only 42 of 53 patients completed a minimum 3-year follow-up and satisfied their inclusion criteria. All the 42 patients included in this analysis showed grade 1 degenerative spondylolisthesis. Of the 42 patients, 22 underwent selective decompression and fusion, and 20 patients underwent multi-segmental decompression and fusion. There was no significant difference in patient baseline demographics (*P* > 0.05) (Table 1). As shown in Table 2, there was no statistical difference in the mean number of surgical decompressed segment between the two groups (*P* > 0.05), while the mean number of the fused segment in group M is more than that in group S (*P* < 0.01). To investigate operative time and intraoperative blood loss, we divided the patients of each group into two subgroups on the basis of the number of surgical segment (two-segment subgroup and three-segment subgroup) and then compared group S with group M in subgroups. The outcomes showed that the mean operative time and intraoperative blood loss in group S were significantly less than those in group M in both subgroups (*P* < 0.01). Two patients in group S presented with postoperative numbness of the lower extremities which was relieved by 2 weeks of nerve nutrition treatment. In group M, three patients had radicular leg pain after surgery, which was relieved by 1 month of symptomatic treatment, another two patient experienced a dural injury and cerebrospinal fluid leakage, which healed spontaneously by means of prolonged drainage (5 days after surgery) and closure of the skin incision with sutures, the other patient had a superficial infection of the incision, which was cured after anti-inflammatory treatment. No other serious complications or reoperation occurred in either group.

**Table 1:** Patient baseline demographic characteristics

	Group S	Group M	*P*
Number of patients	22	20	
Gender (F/M)	10/12	9/11	0.976
Mean age (years)	61.1 ± 6.9	63.0 ± 5.5	0.354
Symptom duration (months)	19.3 ± 12.3	21.6 ± 9.8	0.513
Follow-up (months)	37.6 ± 1.7	38.1 ± 2.4	0.472
Stenosis segment			0.657
L2–5	1	0	
L3–5	9	7	
L4–S1	5	5	
L3–S1	7	8	
Listhesis segment			0.470
L3/4	0	1	
L4/5	14	12	
L5/S1	8	7	

*F* female, *M* male

**Table 2:** Surgical segments, operative time, blood loss, complications, and adjacent segment instability

	Group S	Group M	*P*
NDS	2.36 ± 0.49	2.40 ± 0.50	0.783
NFS	1.18 ± 0.39	2.40 ± 0.50	< 0.01
SSS			0.808
Two-segment	14	12	
Three-segment	8	8	
Operative time (min)			
Two-segment	148.60 ± 11.0	227.5 ± 25.3	< 0.01
Three-segment	180.0 ± 7.6	276.3 ± 14.1	< 0.01
Blood loss (ml)			
Two-segment	292.1 ± 40.6	507.5 ± 55.6	< 0.01
Three-segment	448.8 ± 90.5	731.3 ± 164.6	< 0.01
Complications			
Nerve root injury	2	3	0.656
Wound infection	0	2	0.221
Dural tear	0	1	0.476
ASI	0	3	0.099

*SSS* subgroup based on surgical segment, *NDS* mean number of decompressed segment, *NFS* mean number of fused segment, *ASI* adjacent segment instability

As shown in Table 3, the mean VAS, ODI, and SF-36 scores at 1-year and 3-year follow-up were significantly improved compared with those preoperatively in both groups (*P* < 0.01), but there were no significant differences in these scores between the two groups at each time point (*P* > 0.05).

**Table 3:** Change in VAS, ODI, and SF-36 scores

	Preoperative	1-year follow-up	3-year follow-up
VAS (back)			
Group S	6.1 ± 1.3	2.0 ± 0.6^*^	2.3 ± 0.8^*^
Group M	5.7 ± 1.2	1.9 ± 0.6^*^	2.2 ± 0.8^*^
VAS (leg)			
Group S	7.4 ± 1.5	1.9 ± 0.7^*^	2.2 ± 0.9^*^
Group M	7.7 ± 1.3	2.0 ± 0.7^*^	2.4 ± 0.9^*^
ODI (%)			
Group S	55.5 ± 7.4	22.5 ± 4.2^*^	22.9 ± 4.8^*^
Group M	59.4 ± 7.8	23.8 ± 3.9^*^	25.7 ± 4.9^*^
SF-36			
PF			
Group S	39.3 ± 5.6	56.4 ± 6.2^*^	55.0 ± 6.4^*^
Group M	38.3 ± 6.1	55.0 ± 5.1^*^	53.8 ± 5.6^*^
GH			
Group S	21.4 ± 5.8	40.5 ± 5.5^*^	39.1 ± 6.3^*^
Group M	23.0 ± 5.7	39.5 ± 3.9^*^	38.3 ± 5.7^*^
MH			
Group S	46.0 ± 6.6	58.0 ± 7.4^*^	58.5 ± 6.8^*^
Group M	47.4 ± 6.5	58.4 ± 5.7^*^	59.4 ± 6.0^*^

*PF* physical function, *GH* general health, *MH* mental health

^*^Compared with preoperative, *P* < 0.01

Among patients in both groups, bone fusion was achieved in all the fusion segments at the final follow-up. No hardware loosening or breakage, or fusion cage migration occurred in either group. No iatrogenic spinal instability was noted in the decompressed segments and the adjacent segments in group S. However, in group M, flexion-extension instability in the upper adjacent segment was noted in three patients at 3-year follow-up despite no statistical difference (Table 2, *P* > 0.05).

## Discussion

To date, no consensus exists for the surgical management of MLSS due to lack of evidence to standardize practice, especially the extent of decompression and fusion. In this study, we compared the clinical outcomes of selective decompression and fusion with those of multi-segmental decompression and fusion for the treatment of MLSS.

The so-called selective decompression and fusion in this study refer to precise decompression (decompression was performed only at the responsible sites of the stenotic segments) and selective fusion (fusion was performed only at the slipped segments and segments of potentially iatrogenic spinal instability). Actually, this idea incorporates the concept of “minimal invasion” into the open surgery. The key point is to damage the spinal stable elements as little as possible on the premise of adequate decompression. Based on detailed medical history, careful physical examination, and comprehensive imaging studies, precise decompression of the affected nerve root only at the responsible sites and limited fusion of the slipped or potentially unstable segments were performed, while the extended decompression was not performed.

The present study demonstrated that precise and limited decompression and fusion yielded similar outcomes to multi-segmental decompression and fusion, with shorter operative time, lesser blood loss and trauma, more preservation of motion segments, and fewer complications (although there was no statistically significant difference). An increased extent of decompression and fusion could not contribute to the improvement of clinical efficacy. In agreement with the results, Smorgick et al. [8] reported that decompression and multilevel fusion provided similar clinical outcomes to decompression and single-level fusion in patients with MLSS, with higher operative time and blood loss. Chang et al. [12] also found that the additional fusion did not result in clinical improvements, but with a higher risk of complications over decompression alone for the treatment of lumbar spinal stenosis at 2-year follow-up. Based on the previous studies, we suggest that the key to success for the surgical treatment of MLSS lies in the precision of decompression and fusion, rather than the extent of decompression and fusion.

Although decompression alone was deemed as a feasible treatment with similar outcomes to decompression plus fusion for lumbar spondylolisthesis [13], most studies have suggested that decompression plus fusion for lumbar spondylolisthesis has advantages in improvement of physical health-related quality of life and clinical satisfaction as well as reduction in postoperative leg pain when compared with decompression alone [14–16]. With respect to MLSS, in our opinion, the extent of fixation and fusion need not be identical to the extent of decompression, but should only be confined to the slipped segments and potentially unstable segments after decompression. Lee et al. [17] compared the postoperative lumbar function of patients undergoing long-segment fusion with those undergoing short-segment fusion and found that postoperative ODI scores were significantly worse in the long-segment group than those in the short-segment group. There was no significant difference in the improvement of lower back pain between the two groups. Moreover, the length of the fused segment is positively associated with stress and mobility in the adjacent segments and the incidence of ASD. Yang et al. [18] demonstrated that the incidence of ASD after single-segment, two-segment, and more than three-segment fusion was 11.6%, 14.5%, and 16.3%, respectively. In this study, we noted three patients developed postoperative spinal instability at the upper adjacent segment in group M, which may be an indication of ASD. In addition, it has been reported that long fused segments are correlated with a low fusion rate and a high incidence of implant complications [6, 19], such as implant breakage and loosening, as well as interbody cage migration. Although bony fusion was achieved at all fused segments and no implant complications occurred in our study, unnecessary fusion should be avoided. The small patient population in this study may be the main reason.

Differ from the previous report [9], no iatrogenic instability of the decompressed segments was observed and more spinal motion segments were preserved in group S at the final follow-up. The reasons may be as follows: (1) a proportion of stenotic segments do not have to undergo extensive decompression (a standard total laminectomy and foraminal decompression), adequate and effective decompression of the affected nerve root can be achieved by hemilaminectomy, or undercutting decompression of the lateral recess with care taken to preserve at least 50% of the facet joint. For patients with severe spinal stenosis, laminectomy with preservation of the spinous process and interspinous and supraspinous ligaments was performed instead of the classic full laminectomy in order to preserve the tension of posterior spinal structures, thus minimizing disruption of biomechanical integrity [20, 21]. 2) Care has also been taken to avoid injuring the joint capsule during exposure or screw insertion to protect the facet joint. Special attention should be paid to the fact that a pedicle screw inserted medially will likely damage the cephalic facet joints and increase the risk of ASD [22].

There are several limitations to this study. First, this study is a retrospective analysis with small sample size and short-term follow-up. Prospective multi-center studies with larger sample size and longer follow-up in the future are needed to further determine the benefits of selective decompression and fusion for MLSS. Second, this study did not include the comparison with the non-fused group because limited decompression without fusion is only recommended for elderly, low-demand patients with multiple comorbidities and significant degenerative changes that reduce the likelihood of slip progression. Additionally, the treatment decisions made solely by the senior surgeon may have important implications on clinical outcomes. Despite these points, this study provided valuable data for spine specialists when making clinical decisions in the treatment of MLSS.

## Conclusion

In summary, selective decompression and fusion is a safe and effective method for the treatment of MLSS, with advantages of shorter operative time, less blood loss and trauma, and more preservation of spinal motion segments when compared with multi-segmental decompression and fusion.

## Abbreviations

ASD: Adjacent segment degeneration
GH: General health
MH: Mental health
MLSS: Multi-segment lumbar spinal stenosis with single-segment degenerative spondylolisthesis
ODI: Oswestry Disability Index
PF: Physical function
SF-36: Short Form 36
VAS: Visual analog scale
